# Adsorption of Rare Earths(Ⅲ) Using an Efficient Sodium Alginate Hydrogel Cross-Linked with Poly-γ-Glutamate

**DOI:** 10.1371/journal.pone.0124826

**Published:** 2015-05-21

**Authors:** Shuxia Xu, Zhiwei Wang, Yuqian Gao, Shimin Zhang, Kun Wu

**Affiliations:** College of Life Sciences, Henan Agricultural University, Zhengzhou, 450002, China; Duke University Marine Laboratory, UNITED STATES

## Abstract

With the exploitation of rare earth ore, more and more REEs came into groundwater. This was a waste of resources and could be harmful to the organisms. This study aimed to find an efficient adsorption material to mitigate the above issue. Through doping sodium alginate (SA) with poly-γ-glutamate (PGA), an immobilized gel particle material was produced. The composite exhibited excellent capacity for adsorbing rare earth elements (REEs). The amount of La^3+^ adsorbed on the SA-PGA gel particles reached approximately 163.93 mg/g compared to the 81.97 mg/g adsorbed on SA alone. The factors that potentially affected the adsorption efficiency of the SA-PGA composite, including the initial concentration of REEs, the adsorbent dosage, and the pH of the solution, were investigated. 15 types of REEs in single and mixed aqueous solutions were used to explore the selective adsorption of REEs on gel particles. Scanning electron microscopy (SEM) and Fourier transform infrared (FT-IR) spectroscopy analyses of the SA and SA-PGA gel beads suggested that the carboxyl groups in the composite might play a key role in the adsorption process and the morphology of SA-PGA changed from the compact structure of SA to a porous structure after doping PGA. The kinetics and thermodynamics of the adsorption of REEs were well fit with the pseudo-second-order equation and the Langmuir adsorption isotherm model, respectively. It appears that SA-PGA is useful for recycling REEs from wastewater.

## Introduction

Rare earth elements (REEs), also known as “Industrial Vitamins”, are widely used in numerous technological devices, such as superconductors, magnets, catalysts, and batteries[1]. The amount of REEs mined worldwide increased sharply from 50 kt/year in 1990 to 130 kt/year in 2010[2–4]. With the increasing use of REEs and inappropriate production and post-production treatments, rare earth-related contaminations have emerged in recent years and are leading to a series of environmental problems. For example, a single hybrid car contains approximately 15 kg of REEs[5]. The use of REEs in millions of tons of agricultural fertilizers annually causes the accumulation of these elements in soils[6]. Excessive amounts of REEs have been detected in human bodies due to the consumption of contaminated water. The toxicity of REEs to humans is similar to those of lead, cadmium and other heavy metals. The extraction of REE contaminants from our living environment is a critical challenge and is of great practical significance.

Sodium alginate (SA) is a natural hydrophilic colloidal polysaccharide with an abundance of free carboxyl and hydroxyl groups distributed along its backbone. SA is generally isolated from brown seaweeds and bacteria[7]. Because of its excellent gelation properties, SA has been widely used in the separation of metal ions from solution[8,9]. Other advantages of SA include its stability, good biodegradability and non-toxicity. Interestingly, the gel particles can be formed when meeting the divalent metal ion (Ca^2+^,Ba^2+^), which makes the separation of SA more easy. One problem associated with particles is the low adsorption efficiency.

Poly-γ-glutamate (PGA) is a natural macromolecular polymer that is synthesized by several gram-positive bacteria from the genus *Bacillus*. PGA has unique physicochemical properties, such as film-forming ability, water-retention capacity, plasticity, and biodegradability[10,11]. PGA has been widely used in the fields of pharmaceutical manufacturing, food processing, cosmetics production, protection of plant seeds, and water treatment. PGA is an anionic polypeptide produced via the polymerization of glutamates via γ-amide linkages[12]. Each monomer contains a carboxyl group that can chelate with rare earth ions, resulting in the adsorption of REE ions on PGA. However, because of the water solubility a stabilizer should be used to realize the recovery and separation of PGA-REEs from the liquid.

Previous studies have found that immobilization techniques, such as immobilizing polymers in carriers, can significantly improve the adsorption efficiency of biomaterials [13,14]. In this study, PGA was creatively immobilized in SA gel. After batch experiments of ratio of SA and PGA, SA-PGA particles with a considerable adsorption capacity were produced. The adsorption rate was greater than 87.74% in a fairly high concentration solution of REEs (220 mg/L) and 99.83% in a low concentration solution (60 mg/L). Based on the study of the adsorption mechanism, dilute hydrochloric acid could regenerate the material.

## Materials and Methods

### Chemical reagents

Rare-earth chlorides were purchased from the National Medicine Group Chemical Reagent Co., Ltd. (Shanghai, China). Sodium alginate was purchased from the Xi Long Chemical Co., Ltd. Poly-γ-glutamate powder (1000 kDa) was produced in our laboratory. Other chemicals used in this work were of analytical grade.

### Preparation of the adsorbent

Before the gel preparation, an orthogonal experiment was designed to obtain the optimal formula between sodium alginate and polyglutamic acid(See S1 Text and S1–S3 Tables). 2g of sodium alginate was dissolved in 100 ml of deionized water under vigorous heating and stirring to obtain solution A. After cooling to room temperature, 1 g of poly-γ-glutamate was added to solution A. The solution was then constantly stirred to obtain homogeneous solution B. Next, an appropriate amount of solution B was drawn into a syringe and added to the CaCl_2_ solution (5%, w/w) dropwise at a height of 10 cm (5 drops/sec). Throughout the entire process, the CaCl_2_ solution was constantly stirred with a magnetic stirrer. Then, the gel particles were allowed to stand overnight to harden. Subsequently, excess Ca^2+^ and Na^+^ on the gel surface were removed by washing with a large amount of deionized water. Next, the beads were treated with 0.01% glutaraldehyde solution for 2 hrs. After washing, gels were freeze dried and stored at room temperature. SA gel particles (2%, w/w) were produced using a similar procedure.

### Adsorption experiments

Certain amounts of adsorbent were added into 50ml solution of rare earth ion in 250ml conical flasks, and the pH values of the solution were adjusted to desired values in the range of 2.0–7.0 using 6 M NaOH or 5 M HCl. The samples were agitated for 3 hrs in a rotary shaker at 150 rpm under appropriate temperature. Batch experiments were conducted separately to evaluate the effects of the initial REE concentration, adsorbent dosage, and pH of the solution. Studies on the adsorption kinetics and thermodynamics were conducted using La^3+^ and Ce^3+^ solutions. Samples were collected at specific intervals to measure the residual concentrations of La^3+^ and Ce^3+^. Ca^2+^ would release before the adsorption material saturated in the process of adsorption. These Ca^2+^ had no effect on the detection of residual R^3+^. All of the adsorption experiments were conducted in triplicate, and the average results were presented.

### Determination of REE ions

Inductively coupled plasma-atomic emission spectrometry (ICP-OES, Varian, VISTA—MPXMPX, America) was used to determine the concentrations of the REEs. The operating conditions were as follows: power of 1.2 kW, plasma gas flow rate of 15 L/min, auxiliary gas flow rate of 1.5 L/min, nebulizer pressure of 250 kPa and pump rate of 15 r/min.

### Data analysis

The adsorption capacity (*Q*_*t*_) of the gel particles for REE ions was calculated using the relationship in Eq (1)[15]:

$$Q_{\text{t}}=\frac{C_{i}-C_{t}}{M_{s}}$$ (1)

Additionally, the adsorption rate (*AD*) of REEs from the aqueous solution by the gel particles was calculated using Eq (2):

$$AD=\frac{C_{\text{i}}-C_{t}}{C_{i}}\times 100\%$$ (2)

where *Q*_*t*_ is the adsorption capacity of the gel particles for REEs (mg/g), *C*_*i*_ is the initial REE concentration (mg/L), *C*_*t*_ is the REE concentration in solution at time *t* (mg/L), and *M*_*s*_ is the dry weight of the gel particles (mg/L).

### Adsorption kinetics

The kinetics for the adsorption of REE ions was analyzed using two kinetic models: pseudo-first-order[16] and pseudo-second-order models[17]. Additionally, both of these models have a linear form. The pseudo-first-order Eq (3) and pseudo-second-order Eq (4) are generally expressed as:

$$\mathrm{lg}(Q_{e}-Q_{t})=\mathrm{lg}Q_{e}-k_{1}t$$ (3)

$$\frac{t}{Q_{t}}=\frac{1}{k_{2}Q_{e}{}^{2}}+\frac{t}{Q_{e}}$$ (4)

where *Q*_*e*_ and *Q*_*t*_ (mg/g) are the sorption capacities at equilibrium and at time *t*, respectively, and *k*_*1*_ and *k*_*2*_ are the pseudo-first-order and pseudo-second-order sorption rate constants ((g/mg)/min), respectively.

### Adsorption thermodynamics

The Langmuir and Freundlich isotherm equations are generally used to describe the adsorption thermodynamics. After simplifying the equations, we obtain the following linear forms.

Langmuir isotherm equation:

$$\frac{C_{e}}{Q_{e}}=\frac{C_{e}}{Q_{m}}+\frac{1}{Q_{m}K_{L}}$$ (5)

Freundlich isotherm equation:

$$\ln Q_{e}=\frac{\ln C_{e}}{n}+\ln K_{F}$$ (6)

where *C*_*e*_ is the concentration of REE ions at equilibrium (mg/L), *Q*_*m*_ is the maximum theoretical adsorption amount (mg/g), *K*_*L*_ is the Langmuir adsorption equilibrium constant (L/mg), *K*_*F*_ is the adsorption capacity of the particles (mg/g), and *n* is the affinity between the particles and adsorbate.

## Results and Discussion

### Adsorption conditions

#### Effect of initial concentration of REE ions on the adsorption

**Fig 1:**
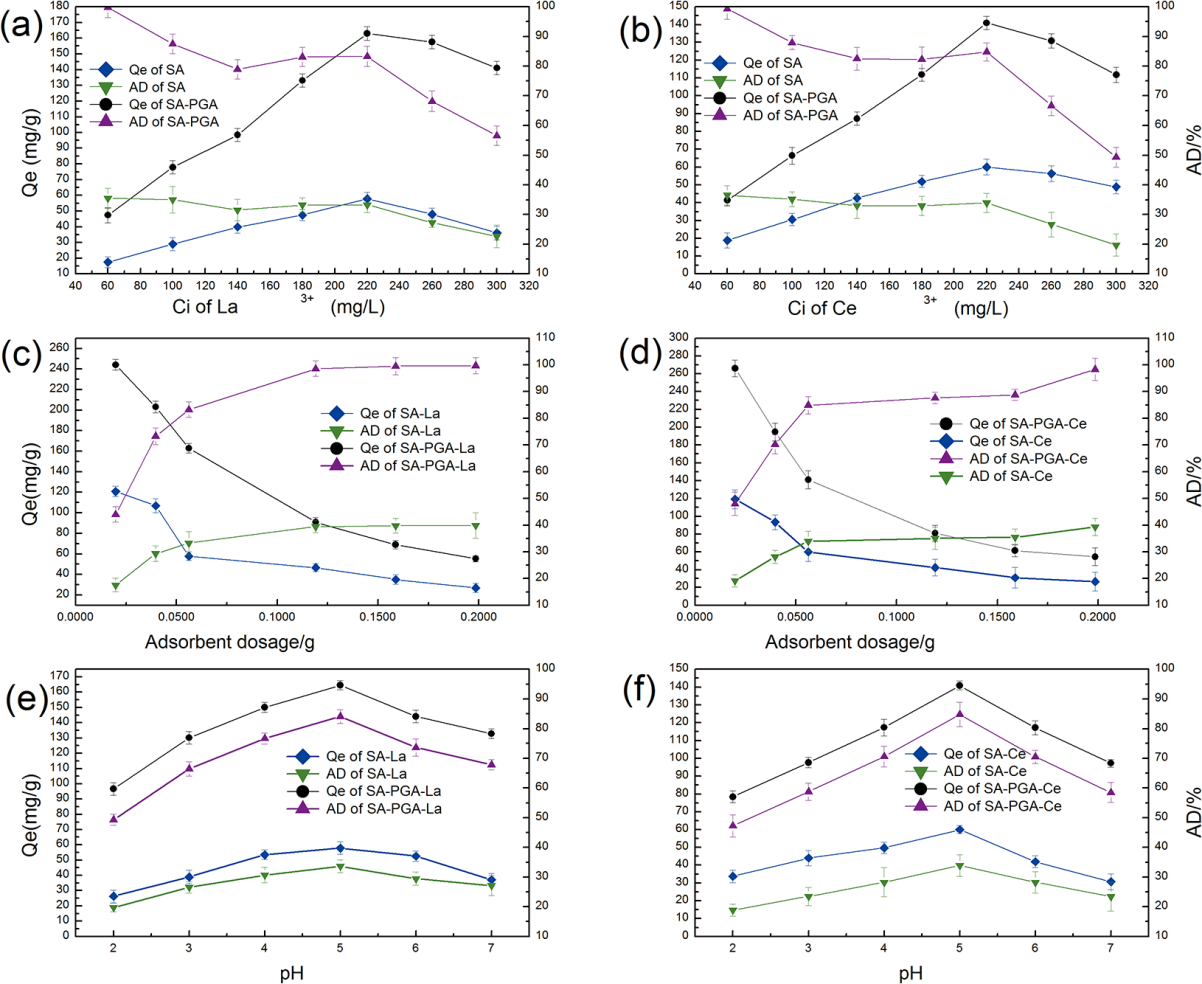
Effects of the initial concentration of REEs, adsorbent dosage, and pH of solution on the adsorption by gel particles (Adsorption conditions: temperature 30°C; 150 rpm; and 3h adsorption time).

The results regarding the effect of initial concentration of REE ions are presented in Fig 1A and 1B. These results indicated that the adsorption of La^3+^ and Ce^3+^ exhibited similar behavior on these two gels. As the ion concentration increased, the adsorption rate decreased after a plateau period. In contrast, the adsorption amount increased in an approximately linear manner before the excess of adsorption sites. Moreover, note that the saturated adsorption amount of SA (57.82 mg/g) was only 35.47% of that of SA-PGA (163.02 mg/g). This result indicated that doping with PGA could greatly improve the efficiency for adsorbing REEs ions.

#### Effect of adsorbent dosage

The effect of adsorbent dosage on the adsorption of REEs is shown in Fig 1C and 1D. The results indicated that adsorption rate improved with the increasing of adsorbent dosage. The adsorption amount decreased with increasing adsorbent dosage. When the adsorbent dosage was greater than 0.0562 g, the adsorption amount obviously decreased. Because there came a large amount of unloaded active sites on the gel beads[18]. At the lowest SA particle dosage, the La^3+^ removal efficiency was 17.62%, whereas at the highest SA particle dosage, the adsorption rate reached 39.88%. In contrast, these two data of SA-PGA were 44.05% and 99.70%, respectively. Thus, this result indicated that SA-PGA particles are a more effective type of adsorbent.

#### Effect of the initial pH

The pH may be the most important parameter in the adsorption process because it can affect not only the surface charge of the adsorbent but also the degree of ionization and the speciation of the adsorbate during the adsorption process[19]. Normally, under low pH conditions, H^+^ will compete with rare earth cations for binding sites. Therefore, it is difficult for REEs to approach the adsorption sites. On the contrary, under basic pH conditions, more sites with negative charges will be exposed toward the adsorbate. Thus, REE ions with positive charges can be adequately attracted. However, under alkaline pH conditions, the REE ions will form hydroxide precipitates. The initial pH was adjusted to pH 2.0–7.0. Fig 1E and 1F show the influence of the initial pH on the adsorption behavior of the gel particles. When pH<3.0, H^+^ had the dominant superiority compared with other cations. The interaction was weak between adsorbent and R^3+^. The concentration of Ca^2+^ reached about 64 mg/L. In pH4.0–5.0, main cations were H^+^, Ca^2+^ and R^3+^. Accompanied by the loss of dominance, Ca^2+^ released and R^3+^ were adsorbed.

#### Mixed adsorption

**Fig 2:**
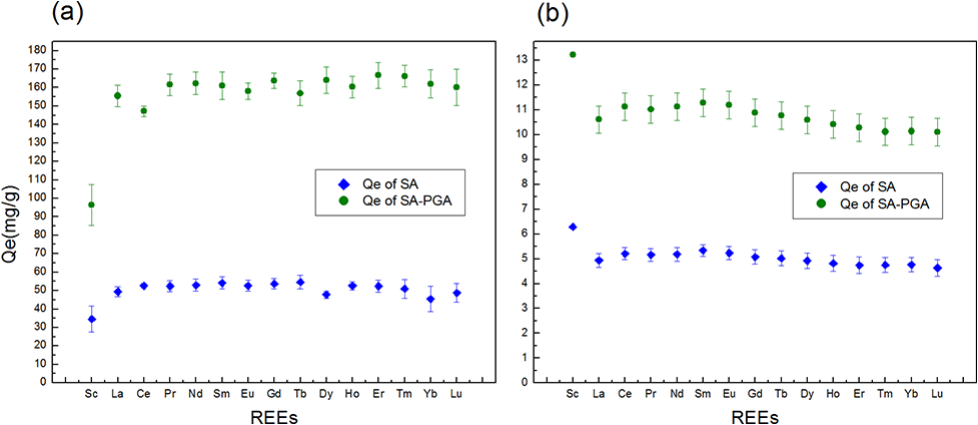
Selective adsorption experiments of REEs: (a) single solution, (b) mixed solution.

Under natural conditions, REEs exist in a mixed state. Therefore, it is necessary to investigate the selectivity of REEs on SA-PGA gel particles. To determine the optimal adsorption conditions, response surface experiments based on single-factor tests were conducted under the following conditions: initial concentration of REEs, 220 mg/L; adsorbent dosage, 0.0562 g; pH 5.0; 150 rpm; and 30°C. In the single solution of 15 rare earth elements (Fig 2A), the adsorption amount for scandium was obviously smaller than those for the other rare earths. Because the essence of adsorption is electrical neutralization between negative and positive ions. The number of active sites combined cations is limited. But the relative atomic mass of Sc is much smaller than other rare earth. The selective difference among the 15 types of elements was not significant. In the mixed adsorption(Fig 2B), these was a weak trend: adsorption amount of light rare earth was more than heavy rare earths. The high adsorption of Sc might result from the competition between Sc and adsorption sites was stronger than other rare earth ions in mixture solution. Comparing the two types of adsorption, the adsorption amount in the single solution was approximately equal to the sum of the adsorption amount of each element in the single solution. This material was more suitable for enrichment and recovery of mixed rare earth ions.

### Material characterization

#### Infrared Analysis

**Fig 3:**
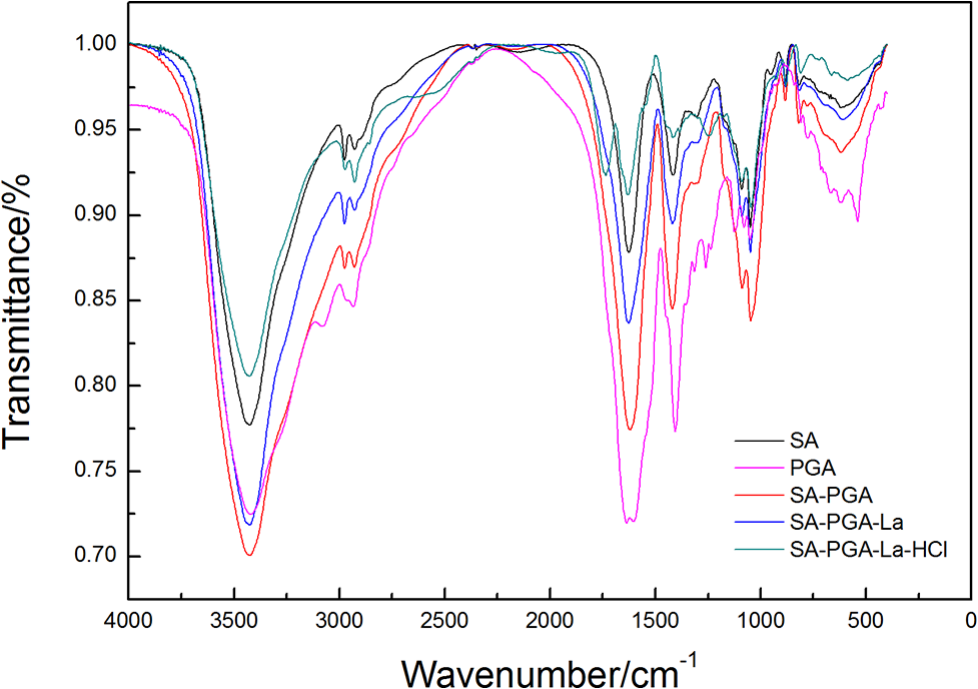
FT-IR spectra of SA, PGA, SA-PGA, SA-PAG-La, and SA-PGA-La-HCl gels.

The FT-IR spectroscopy (Bruker Spectrophotometer TENSOR 27, Germany) results are shown in Fig 3. Some characteristic vibrational modes of SA are 3426 cm^-1^ *ν*_(C-OH)_ and 1626cm^-1^ *ν*_as(COO-)_, which are assigned to stretching vibrations of the carboxyl group[20]. There are two new absorption peaks attributed to 1615cm^-1^ δ(-OH)+ν(COO^-^)_asym_ and 1419cm^-1^ ν(COO^-^)_sym_ in SA-PGA. Under normal circumstance, absorption peak of carbonyl _(in COOH)_ should appear at 1700–1725cm^-1^. From the view of coordination chemistry, the type of carboxyl in SA-PGA gel was a single-tooth ligand with Ca^2+^. Table 1 shows the main vibrational modes for adsorbents. Compared with the SA spectrum, that of SA-PGA gel showed a new absorption peak at 1126cm^-1^ attributed to ν_(CN)_. This suggested that PGA was successfully immobilized in the SA gel. The other peaks of the SA-PGA gel exhibited no difference with respect to those of the SA gel, which indicated that PGA was very likely fixed through a mode of non-chemical interaction, such as electrostatic attraction or hydrogen bonding[21]. After the adsorption of La^3+^, the peak at 1615 cm^-1^ attributed to δ_(-OH)_+ν_(COO-)asym_ had shifted to a stronger wavenumber 1629cm^-1^. It showed that carboxyl in SA and PGA participated in the coordination with REEs ions. Moreover, 1733cm^-1^ assigned to ν_(C = O in COOH)_ absorption peak only appeared in SA-PGA-La-HCl spectrum. It showed that free-COOH transformed into-COOCa in the process of preparing SA-PGA gel. This result suggested that the carboxyl group coordinated with the rare earth ions both in SA and PGA. The adsorption mechanism was likely to be cation exchange between-COOCa and R^3+^.

**Table 1:** Assignment of the main vibrational modes.(C_HCl_:0.1mol/L,50mL).

Vibration	ν(OH) OR ν(NH)	ν(CH)	ν(C = O _in COOH_)	δ(-OH)+ν(COO-)asym	ν(COO-)sym	δ(CCH)+δ (OCH)	ν(CN)	ν(CO)	C1-H deformation mannuronic acid residues	Mannuronic acid residues
SA	3426.00	2926.08		1626.20	1415.84	1304.90		1048.78	882.46	817.26
PGA	3427.38	2932.50		1611.70	1407.73		1126.85	1042.42		
SA-PGA	3425.39	2927.47		1615.82	1419.41	1304.07	1088.34	1047.45	883.00	818.89
SA-PGA-La	3425.41	2926.57		1629.02	1418.88	1293.20	1088.84	1048.74	882.13	821.00
SA-PGA-La-HCl	3427.61	2926.45	1733.96	1630.39	1417.69	1247.92	1066.94	1046.49	879.31	805.96

#### Morphology

**Fig 4:**
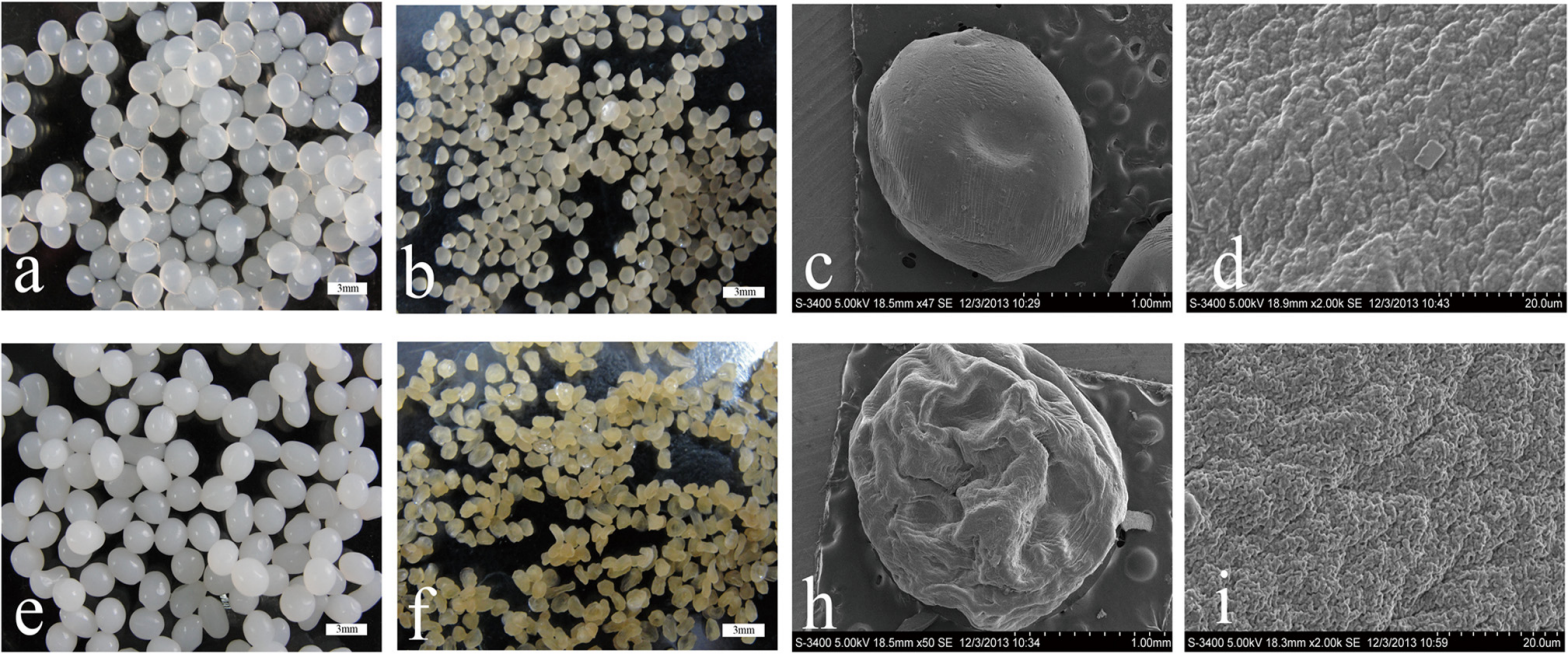
Digital photographs of SA gels (a, b, c, d) and SA-PGA gels (e, f, h, i).

Distinct differences were observed in the morphologies between SA and SA-PGA gel beads (Fig 4). Fig 4A and 4E show the shapes of wet gel particles. Compared with the SA, SA-PGA had a larger size because of doping with poly-γ-glutamate(average diameter 1mm). The color of SA gel appeared semitransparent, but the SA-PGA particles were milk white. After freeze drying, the color of SA-PGA gel darkened. Fig 4C, 4D, 4H and 4I present SEM images (Hitachi S3400N SEM, Japan). Under 2000x magnification, SA-PGA exhibited a uniform porous structure, whereas the structure of SA appeared compact and non-porous. Consequently, SA-PGA had a larger specific surface area, which was beneficial to improve the adsorption efficiency.

### Kinetic model

**Fig 5:**
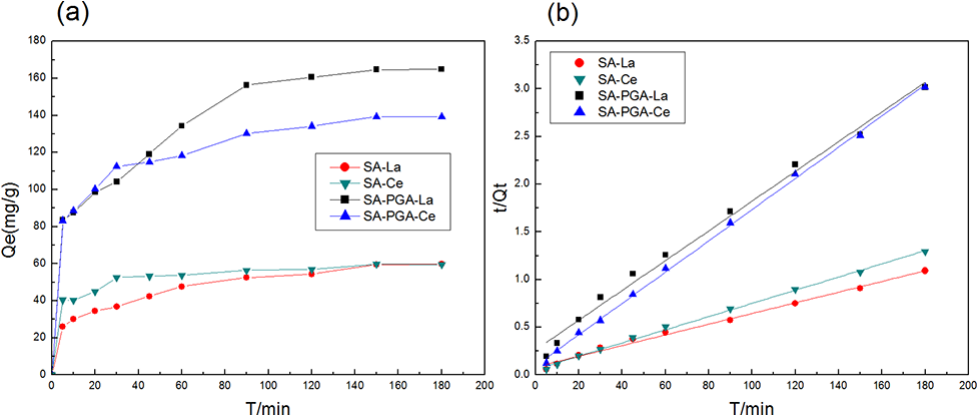
Adsorption kinetic curves (a) and pseudo-second-order plots (b) for the adsorption by gel particles.

Fig 5 presents the kinetic data of these two adsorbents under an initial REE ion concentration of 220 mg/L. As shown in Fig 5A, the adsorption process was rapid during the first 5 min and continued with a slower rate from 5–90 min, and then it reached adsorption equilibrium. To investigate the amount of adsorbed REEs, the kinetics for the adsorption of REEs by gel particles was modeled using pseudo-first-order and pseudo-second-order kinetics equations.

The characteristic parameters and regression coefficients (*r* ^*2*^) of the models were determined by linear regression analysis and are listed in Table 2. According to the linear pseudo-second-order plot obtained by plotting *t/Q*_*t*_ vs. t (in Fig 5B) and the *r* ^*2*^ value, adsorption on the gel was successfully described by the pseudo-second-order model.

**Table 2:** Pseudo-first-order and pseudo-second-order kinetic parameters for the adsorption of REE ions. (C_0_: 220 mg/L).

REEs		Pseudo-first-order model			Pseudo-second-order model		
	Q_e_(exp)(mg/g)	k_1_ (/min)	Q_e_ (mg/g)	r^2^	k_2_ ((g/mg)/min)	Q_e_ (mg/g)	r^2^
SA-PGA-La	160.39	0.0253	95.94	0.8903	0.0003	166.67	0.9929
SA-La	59.67	0.0138	34.04	0.7896	0.0009	62.50	0.9920
SA-PGA-Ce	139.23	0.0207	61.94	0.7777	0.0008	142.86	0.9978
SA-Ce	59.63	0.0184	19.45	0.9448	0.0027	62.50	0.9989

### Thermodynamic model

#### Adsorption isotherms

**Fig 6:**
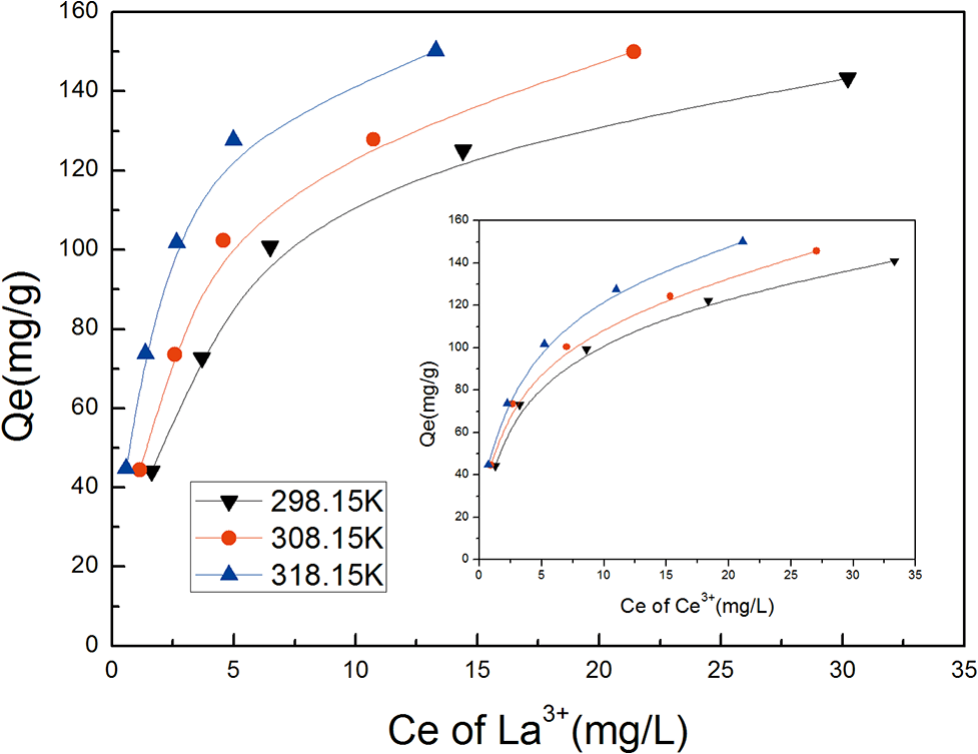
Adsorption isotherms of La^3+^ and Ce^3+^ on SA-PGA gel particles.

The equilibrium adsorption isotherm is one of the most important parameters for understanding the mechanism of the adsorption system[22]. It describes the relationship between *Q*_*e*_ and *C*_*e*_ at a certain temperature. In this work, temperatures of 298.15 K, 308.15 K, and 318.15 K were selected to investigate the adsorption of REE ions. Additionally, the corresponding adsorption isotherms were plotted. The results are presented in Fig 6.

Traditionally, the adsorption process can be described using the Langmuir and the Freundlich isotherms. The Langmuir model assumes that adsorption occurs at specific homogenous sites within the adsorbent, and it has been successfully applied in many monolayer adsorption studies[23]. The Freundlich model assumes a heterogeneous adsorption surface with sites that have different energies of adsorption and that are not equally available[24].

From the experimental data, the adsorption equations were obtained using Eqs (5) and (6). The isotherm constants and correlation coefficients (*r* ^*2*^) were calculated by plotting *C*_*e*_ */Q*_*e*_ vs. *Ce* and *ln Q*_*e*_ vs. ln Ce and are presented in Table 3. The *K*_*L*_ values are the key parameters for calculating the thermodynamics constants. Additionally, it is generally stated that values of n ranged from 2to 10 representing good adsorption. The results indicated that the values of n were all greater than 2, which implied that REE ions were favorably adsorbed by the gel. By comparing the adsorption equilibrium constants and the adsorption affinities of these two types of materials, it was observed that the combination with poly-γ-glutamate was beneficial in enhancing the adsorption efficiency. Through the combination of internal structure and composition, the thermodynamic features of SA-PGA exhibited superior mass transfer ability.

**Table 3:** Isotherm constants and correlation coefficients for the adsorption of REEs on gel particles at different temperatures.

REEs		Langmuir isotherm						Freundlich isotherm					
	T/K	Q_m_ (mg/g)		K_L_(L/mg)		r^2^		K_F_(mg/g)		n		r^2^	
		SA-PGA	SA	SA-PGA	SA	SA-PGA	SA	SA-PGA	SA	SA-PGA	SA	SA-PGA	SA
La	298.15	163.93	81.97	0.2276	0.0462	0.9997	0.9975	41.1243	7.3272	2.4969	1.8539	0.9387	0.9683
Ce	298.15	153.85	90.91	0.2539	0.0303	0.9958	0.9925	44.5316	5.5639	2.8910	1.6964	0.9733	0.9920
La	308.15	172.41	84.03	0.2974	0.0619	0.9991	0.9992	47.5271	9.2424	2.4522	1.9320	0.9476	0.9788
Ce	308.15	158.73	81.97	0.3043	0.0644	0.9932	0.9952	48.9207	9.6688	2.9019	2.004	0.9846	0.9799
La	318.15	178.57	80.65	0.5490	0.0965	0.9997	0.9987	62.7965	12.4336	2.4832	2.1468	0.9454	0.9802
Ce	318.15	166.67	75.19	0.3636	0.1254	0.9944	0.9939	53.2821	14.8530	2.7894	2.4510	0.9885	0.9908

#### Calculation of the thermodynamic constants

In the present study, the thermodynamics of the adsorption process were investigated in terms of the Gibbs free energy (*ΔG*), enthalpy (*ΔH*), and entropy (*ΔS*) [25]. The effect of temperature on the adsorption process was studied at three temperatures (298.15 K, 308.15 K, and 318.15 K) and five different concentrations of REE ions. *ΔH* and *ΔS* were calculated from the slope and intercept of the linear plot of *ln (Qe/Ce)* vs. *1/T* according to Eq (7), i.e., the Van't Hoff equation. The values of *ΔG* were calculated using Eq (8).

$$\ln\frac{Q_{e}}{C_{e}}=-\frac{\Delta G}{RT}=-\frac{\Delta H}{RT}+\frac{\Delta S}{R}$$ (7)

$$\Delta G=-RT\ln\frac{Q_{e}}{C_{e}}$$ (8)

The thermodynamic constants are shown in Table 4. Because all of the values of *ΔG* were negative, the process could occur spontaneously at room temperature. The absolute values of *ΔG* increased with increasing temperature. Additionally, these values showed that the increasing temperature improved the feasibility of the adsorption process[26]. Moreover, the absolute values of SA-PGA were considerably larger than those of SA. This result indicated that after cross-linking of poly-γ-glutamate, the adsorption capacity had greatly increased. Because all the values of *ΔH* were positive, the adsorption was an endothermic process. In other words, the higher the temperature of the system, the better the result. Viewed from values of *ΔH* (20.1~418.4 kJ/mol), the process was more inclined to chemical adsorption[27]. The degrees of freedom on the liquid-solid surface increased during the adsorption process. This increase might be because the solute molecules lost some of their degrees of freedom during the process of exchanging from the liquid phase to the solid-liquid interface. At the same time, the release of water molecules restored the state of relative freedom. Therefore, it caused the entropy to increase and eventually led to *ΔS*>0.

**Table 4:** Thermodynamic parameters for the adsorption of REEs on the gel particles.

REE ion concentration (mg L^−1^)						ΔG (kJ/mol) at temperature					
	REEs	ΔH (kJ/mol)		ΔS((J/mol)/K)		298.15 K		308.15 K		318.15 K	
		SA-PGA	SA	SA-PGA	SA	SA-PGA	SA	SA-PGA	SA	SA-PGA	SA
60	La	40.9797	32.1087	164.4509	115.9803	-8.1942	-2.5314	-9.3893	-3.5537	-11.5034	-4.8585
60	Ce	23.4621	17.7614	108.2483	65.3480	-8.7987	-1.7115	-9.9242	-2.4265	-10.9618	-3.0163
100	La	39.6744	22.6640	157.3840	83.4726	-7.3888	-2.2282	-8.5895	-3.0470	-10.5544	-3.8985
100	Ce	14.9985	7.5062	76.2477	29.3700	-7.7327	-1.2623	-8.4993	-1.5186	-9.2576	-1.8514
140	La	36.2324	23.8113	143.9985	84.8028	-6.8022	-1.5279	-7.9636	-2.2414	-9.6959	-3.2310
140	Ce	20.6603	5.8634	89.4586	21.9956	-6.0583	-0.7004	-6.8216	-0.9020	-7.8536	-1.1412
180	La	43.7649	17.6506	164.0352	62.5629	-5.3574	-0.9859	-6.3468	-1.6503	-8.6674	-2.2354
180	Ce	21.9656	7.7920	89.2092	26.5017	-4.6965	-0.1055	-5.3656	-0.3828	-6.4905	-0.6350
220	La	35.6754	16.9107	132.3589	57.2835	-3.8603	-0.1413	-4.9852	-0.7896	-6.5170	-1.2838
220	Ce	20.3859	5.6145	80.2966	18.8811	-3.5778	-0.0224	-4.3152	-0.1877	-5.1868	-0.4010

### Desorption experiments

**Fig 7:**
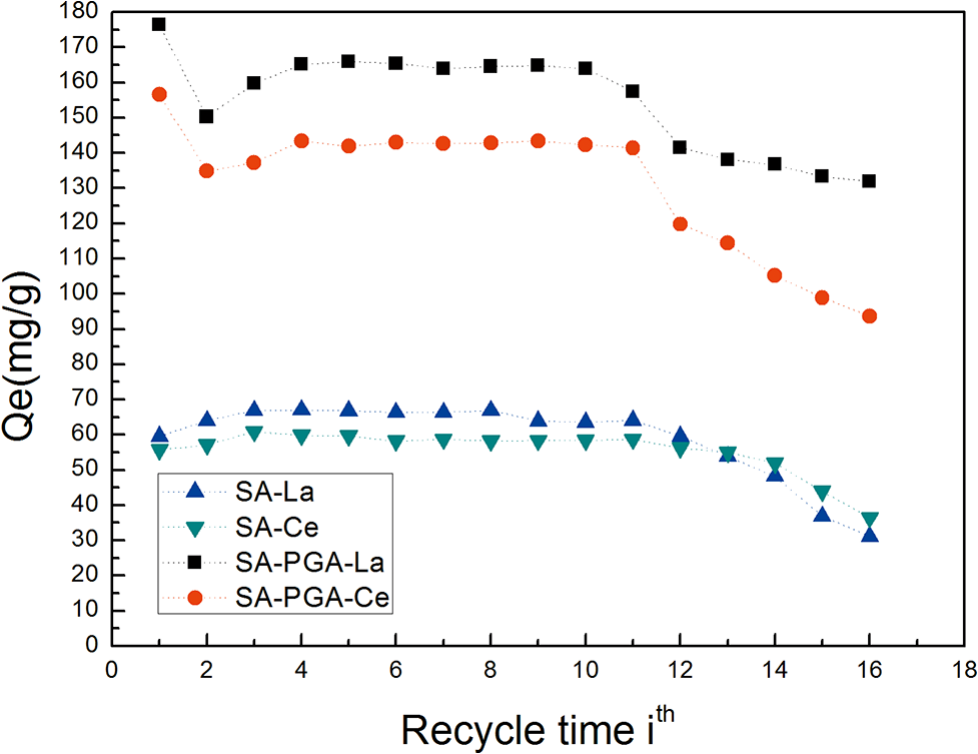
Regeneration of these two gel particles (Adsorption conditions: 30 ml of 0.05 M hydrochloric acid solution, 30 min, 150 rpm, 30°C).

For the enrichment or circulation of REEs, desorption is another key issue. In this work, 0.05 M hydrochloric acid solution was used as the eluent. Additionally, the desorption rate was greater than 99.00%. During the first ten adsorption-desorption cycles, these two adsorbents could retain their adsorption capacities (Fig 7). However, their adsorption capacities subsequently began to slowly decrease. During the experiments, the hydrochloric acid served not only as the desorption agent but also as the modifier that could help to improve the adsorption ability. Due to the release of Ca^2+^ during the adsorption-desorption process[28], the corresponding binding sites were replaced by the R^3+^. Additionally, a new bright silver metallic luster appeared on the surface of the adsorbents.

## Conclusions

This study showed that SA-PGA gel particles possessed a considerable adsorption capacity for REEs(Ⅲ). High or low pH conditions inhibited adsorption. From Sc to Lu, adsorbent showed good adsorption on them. The pseudo-second-order kinetic model agreed well with the dynamic adsorption. The thermodynamic parameters indicated that the adsorption was an endothermic process and could occur spontaneously. The adsorption mechanism might be cation exchange between—COOH and R^3+^ according to FTIR. The SA-PGA particle exhibited a preferential effect on both high and low concentrations of REEs. It still maintained good adsorption capability after 10 adsorption-desorption cycles. SA-PGA can be potentially used in recycling of REEs.

## Supporting Information

S1 Text Effect of ratios of PGA to SA on REE adsorption. (DOCX)

S1 Table Levels of factors in orthogonal experiment (%, w/w). (DOCX)

S2 Table Design and results of orthogonal experiment. (DOCX)

S3 Table Variance analysis of La^3+^ adsorption on SA-PGA. (DOCX)

## References

[1] Moriwaki H, Koide R, Yoshikawa R, Warabino Y, Yamamoto H (2013) Adsorption of rare earth ions onto the cell walls of wild-type and lipoteichoic acid-defective strains of Bacillus subtilis. Applied microbiology and biotechnology 97: 3721–3728. 10.1007/s00253-012-4200-3 22684329

[2] Chen ZH (2011) Global rare earth resources and scenarios of future rare earth industry. Journal of rare earths 29: 1–6.

[3] Gordon B. Haxel JBH, and Orris G (2002) Rare earth elements: critical resources for high technology. Available: http://pubs.usgs.gov/fs/2002/fs087-02/

[4] Studies CfSaI (2010) Rare earth elements: a wrench in the supply chain. Center for Strategic and International Studies.Available: http://csis.org/files/publication/101005_DIIG_Current_Issues_no22_Rare_earth_elements.pdf

[5] Brioschi L, Steinmann M, Lucot E, Pierret M-C, Stille P, Prunier J, et al (2013) Transfer of rare earth elements (REE) from natural soil to plant systems: implications for the environmental availability of anthropogenic REE. Plant and soil 366: 143–163.

[6] Bremmer W (1994) Rare earth applications in Chinese agriculture elements. Rare Earths Specialty Metals Appl Technol 3: 20–24.

[7] Windhues T, Borchard W (2003) Effect of acetylation on physico-chemical properties of bacterial and algal alginates in physiological sodium chloride solutions investigated with light scattering techniques. Carbohydrate polymers 52: 47–52.

[8] Yu K, Ho J, McCandlish E, Buckley B, Patel R, Li Z, et al (2013) Copper ion adsorption by chitosan nanoparticles and alginate microparticles for water purification applications. Colloids and Surfaces A: Physicochemical and Engineering Aspects 425: 31–41.

[9] Kwiatkowska-Marks S WMaKL (2011) Biosorption of heavy metals on alginate beads. Przem Chem 90: 1924–1930.

[10] Taniguchi M, Kato K, Shimauchi A, Xu P, Fujita K-I, Tanaka T, et al (2005) Physicochemical properties of cross-linked poly-γ-glutamic acid and its flocculating activity against kaolin suspension. Journal of bioscience and bioengineering 99: 130–135. 16233769 10.1263/jbb.99.130

[11] Mark SS, Crusberg TC, DaCunha CM, Iorio AAD (2006) A Heavy Metal Biotrap for Wastewater Remediation Using Poly-γ-Glutamic Acid. Biotechnology progress 22: 523–531. 16599572 10.1021/bp060040s

[12] Ashiuchi M, Misono H (2002) Biochemistry and molecular genetics of poly-γ-glutamate synthesis. Applied Microbiology and Biotechnology 59: 9–14. 12073126 10.1007/s00253-002-0984-x

[13] Ngomsik A-F, Bee A, Siaugue J-M, Talbot D, Cabuil V, Cote G (2009) Co (II) removal by magnetic alginate beads containing Cyanex 272. Journal of hazardous materials 166: 1043–1049. 10.1016/j.jhazmat.2008.11.109 19157703

[14] Zhang L, Wu DM, Zhu BH, Yang YH, Wang L (2011) Adsorption and selective separation of neodymium with magnetic alginate microcapsules containing the extractant 2-ethylhexyl phosphonic acid mono-2-ethylhexyl ester. Journal of Chemical & Engineering Data 56: 2280–2289.

[15] Demirbas E, Kobya M, Senturk E, Ozkan T (2004) Adsorption kinetics for the removal of chromium (VI) from aqueous solutions on the activated carbons prepared from agricultural wastes. Water Sa 30: p. 533–539.

[16] Lagergren S (1898) About the theory of so-called adsorption of solution substances.

[17] Ho Y, McKay G (1998) A two-stage batch sorption optimized design for dye removal to minimize contact time. Process Safety and Environmental Protection 76: 313–318.

[18] Shukla A, Zhang Y-H, Dubey P, Margrave J, Shukla SS (2002) The role of sawdust in the removal of unwanted materials from water. Journal of Hazardous Materials 95: 137–152. 12409244 10.1016/s0304-3894(02)00089-4

[19] Bao WW, Zou HF, Gan SC, Xu XC, Ji GJ, Zheng KY (2013) Adsorption of heavy metal ions from aqueous solutions by zeolite based on oil shale ash: Kinetic and equilibrium studies. Chemical Research in Chinese Universities 29: 126–131.

[20] Shu T, Lu P, He N (2013) Mercury adsorption of modified mulberry twig chars in a simulated flue gas. Bioresource technology 136: 182–187. 10.1016/j.biortech.2013.02.087 23567680

[21] Chen H, Zhao J, Dai GL (2011) Silkworm exuviae—A new non-conventional and low-cost adsorbent for removal of methylene blue from aqueous solutions. Journal of Hazardous materials 186: 1320–1327. 10.1016/j.jhazmat.2010.12.006 21185648

[22] Chen H, Zhao J, Wu JY, Dai GL (2011) Isotherm, thermodynamic, kinetics and adsorption mechanism studies of methyl orange by surfactant modified silkworm exuviae. Journal of hazardous materials 192: 246–254. 10.1016/j.jhazmat.2011.05.014 21612865

[23] Langmuir I (1918) The adsorption of gases on plane surfaces of glass, mica and platinum. Journal of the American Chemical society 40: 1361–1403.

[24] Freundlich H (1906) Über die adsorption in lösungen. Zeitschrift für Physikalische.

[25] Diban N, Ruiz G, Urtiaga A, Ortiz I (2008) Recovery of the main pear aroma compound by adsorption/desorption onto commercial granular activated carbon: Equilibrium and kinetics. Journal of food engineering 84: 82–91.

[26] Sari A, Tuzen M (2008) Biosorption of total chromium from aqueous solution by red algae (Ceramium,virgatum): Equilibrium, kinetic and thermodynamic studies. Journal of hazardous materials 160: 349–355. 10.1016/j.jhazmat.2008.03.005 18406520

[27] Kiliç M, Yazici H, Solak M (2009) A comprehensive study on removal and recovery of copper (II) from aqueous solutions by NaOH-pretreated Marrubium globosum ssp. globosum leaves powder: Potential for utilizing the copper (II) condensed desorption solutions in agricultural applications. Bioresource technology 100: 2130–2137. 10.1016/j.biortech.2008.11.002 19097885

[28] Wang FC, Zhao JM, Pan F, Zhou HC, Yang XF, Li WS, et al (2013) Adsorption properties toward trivalent rare earths by alginate beads doping with silica. Industrial & Engineering Chemistry Research 52: 3453–3461.

