# Supplementary material for: Adsorption of Rare Earths(Ⅲ) Using an Efficient Sodium Alginate Hydrogel Cross-Linked with Poly-γ-Glutamate
Source: PLoS One. 2015 May 21;10(5):e0124826. doi: 10.1371/journal.pone.0124826 (PMC4440748; doi:10.1371/journal.pone.0124826)
Supplement: S1 Text — (DOCX) [file pone.0124826.s001.docx]

**Supporting information**

**S1 Text.** **Effect of ratios of PGA to SA on REE adsorption.**

Based on the single factor experiment, the ratios of PGA to SA and the concentration of CaCl_2_ and Glutaraldehyde were chosen as the influence factors of an orthogonal experiment. Adsorption rate of REEs was chosen as the main evaluation standards. At the same time, we also considered the spheronization of particles, mechanical strength and the resistance with acid. Various levels of factors were shown in S1 Table.

The results of orthogonal experiment was shown in the S2 and S3 Tables. It could be seen that the experimental group 5 (SA 2%, PGA 1%, calcium chloride 5%, glutaraldehyde 0.1%) had the best adsorption efficiency. At the same time, there were suitable mechanical strength and acid resistance (pH 2.0, HCl). The range analysis showed that the order of impacting factors on the adsorption was calcium chloride > SA> PGA > glutaraldehyde. The particles gotten from group5 had good physicochemical characteristic: good-sphericity, high mechanical strength, better immobilization and enough active sites. The mass transfer resistance and immobilization reached an excellent equilibrium.
