# Supplementary material for: Adsorption of Rare Earths(Ⅲ) Using an Efficient Sodium Alginate Hydrogel Cross-Linked with Poly-γ-Glutamate
Source: PLoS One. 2015 May 21;10(5):e0124826. doi: 10.1371/journal.pone.0124826 (PMC4440748; doi:10.1371/journal.pone.0124826)
Supplement: S1 Table — (DOCX) [file pone.0124826.s002.docx]

**Supporting information**

**S1 Table. Levels of factors in orthogonal experiment (%, w/w).**

| Level | Factor | | | |
| --- | --- | --- | --- | --- |
|  | SA/% | PGA/% | CaCl_2_/% | Glutaraldehyde/% |
| 1 | 1 | 0.5 | 3 | 0.1 |
| 2 | 2 | 1 | 4 | 0.2 |
| 3 | 3 | 1.5 | 5 | 0.3 |
