# Supplementary material for: Adsorption of Rare Earths(Ⅲ) Using an Efficient Sodium Alginate Hydrogel Cross-Linked with Poly-γ-Glutamate
Source: PLoS One. 2015 May 21;10(5):e0124826. doi: 10.1371/journal.pone.0124826 (PMC4440748; doi:10.1371/journal.pone.0124826)
Supplement: S2 Table — (DOCX) [file pone.0124826.s003.docx]

**Supporting information**

**S2 Table Design and results of orthogonal experiment.**

| Group | Factors | | | | La/% | Mechanical strength | | Acid-resistance | |
| --- | --- | --- | --- | --- | --- | --- | --- | --- | --- |
|  | A | B | C | D |  |  | |  | |
| 1 | 1 | 0.5 | 3 | 0.1 | 80.94 | poor | | poor | |
| 2 | 1 | 1 | 4 | 0.2 | 89.94 | poor | | poor | |
| 3 | 1 | 1.5 | 5 | 0.3 | 97.58 | poor | | poor | |
| 4 | 2 | 0.5 | 4 | 0.3 | 93.59 | moderate | | nice | |
| 5 | 2 | 1 | 5 | 0.1 | 99.51 | nice | | nice | |
| 6 | 2 | 1.5 | 3 | 0.2 | 93.02 | nice | | moderate | |
| 7 | 3 | 0.5 | 5 | 0.2 | 95.30 | nice | | moderate | |
| 8 | 3 | 1 | 3 | 0.3 | 95.64 | nice | | moderate | |
| 9 | 3 | 1.5 | 4 | 0.1 | 96.32 | moderate | | moderate | |
| K_1_ | 89.487 | 89.943 | 89.867 | 92.257 |  |  |  | |  |
| K_2_ | 95.373 | 95.030 | 93.283 | 92.753 |  |  |  | |  |
| K_3_ | 95.753 | 95.640 | 97.453 | 95.603 |  |  |  | |  |
| R | 6.266 | 5.697 | 7.596 | 3.346 |  |  |  | |  |
