# Supplementary material for: Adsorption of Rare Earths(Ⅲ) Using an Efficient Sodium Alginate Hydrogel Cross-Linked with Poly-γ-Glutamate
Source: PLoS One. 2015 May 21;10(5):e0124826. doi: 10.1371/journal.pone.0124826 (PMC4440748; doi:10.1371/journal.pone.0124826)
Supplement: S3 Table — (DOCX) [file pone.0124826.s004.docx]

**Supporting information**

**S3 Table Variance analysis of La^3+^ adsorption on SA-PGA.**

| Source of variance | Degree of freedom | Sum of square | F |
| --- | --- | --- | --- |
| SA | 2 | 74.086 | 1.239 |
| PGA | 2 | 58.698 | 0.982 |
| CaCl_2_ | 2 | 86.855 | 1.452 |
| Glutaraldehyde | 2 | 19.569 | 0.327 |
| Error | 2 | 22.310 |  |
| Total | 10 | 261.581 |  |
